# EphrinA1-Fc attenuates myocardial ischemia/reperfusion injury in mice

**DOI:** 10.1371/journal.pone.0189307

**Published:** 2017-12-13

**Authors:** Augustin DuSablon, Justin Parks, K’Shylah Whitehurst, Heather Estes, Robert Chase, Eleftherios Vlahos, Uma Sharma, David Wert, Jitka Virag

**Affiliations:** Department of Physiology, Brody School of Medicine, East Carolina University, Greenville, North Carolina, United States of America; Emory University, UNITED STATES

## Abstract

EphrinA1, a membrane-bound receptor tyrosine kinase ligand expressed in healthy cardiomyocytes, is lost in injured cells following myocardial infarction. Previously, we have reported that a single intramyocardial injection of chimeric ephrinA1-Fc at the time of ischemia reduced injury in the nonreperfused myocardium by 50% at 4 days post-MI by reducing apoptosis and inflammatory cell infiltration. In a clinically relevant model of acute ischemia (30min)/reperfusion (24hr or 4 days) injury, we now demonstrate that ephrinA1-Fc reduces infarct size by 46% and completely preserves cardiac function (ejection fraction, fractional shortening, and chamber dimensions) in the short-term (24hrs post-MI) as well as long-term (4 days). At 24 hours post-MI, diminished serum inflammatory cell chemoattractants in ephrinA1-Fc-treated mice reduces recruitment of neutrophils and leukocytes into the myocardium. Differences in relative expression levels of EphA-Rs are described in the context of their putative role in mediating cardioprotection. Validation by Western blotting of selected targets from mass spectrometry analyses of pooled samples of left ventricular tissue homogenates from mice that underwent 30min ischemia and 24hr of reperfusion (I/R) indicates that ephrinA1-Fc administration alters several regulators of signaling pathways that attenuate apoptosis, promote autophagy, and shift from FA metabolism in favor of increased glycolysis to optimize anaerobic ATP production. Taken together, reduced injury is due a combination of adaptive metabolic reprogramming, improved cell survival, and decreased inflammatory cell recruitment, suggesting that ephrinA1-Fc enhances the capacity of the heart to withstand an ischemic insult.

## Introduction

Coronary heart disease is the leading cause of cardiovascular deaths nationally and globally. Every year, in the United States alone 1 in 7 people that suffer from a heart attack die each year at an annual cost of $316 billion dollars in direct expenditures and productivity losses nationally and globally, this exceeds 17.3 million people and over $863 billion[1]. Occlusion of a coronary artery due to the rupture of an unstable plaque is the most common cause of MI [2]. Myocardial necrosis begins within 40 min of coronary occlusion and becomes transmural within 14 h [3, 4]. Structural remodeling of the myocardial architecture in both the infarcted area and non-infarcted area leads to progressive ventricular dilation, dysfunction, and a decline in systolic function that eventually results in irreversible heart failure [4–7].

In addition, the heart is subject to global ischemia with possible subsequent reperfusion injury during many cardiac surgical procedures such as coronary artery bypass graft (CABG). The incidence of some degree of new myocardial necrosis ranges between 40% and 60% following CABG [8]. Reperfusion therapy has been the standard of care to reduce acute ischemic injury, preserve left ventricular (LV) function, and reduce mortality since 1986. This therapy must be applied within hours of the occlusive event and, paradoxically, it can worsen oxidative injury [9, 10]. Its benefits have reached their outer limits, and agents currently used to slow the progressive remodeling increase long-term survival but do not restore normal cardiac function. The CAESAR consortium, developed by NIH in 2011, is using a rigorous multicenter, randomized model to identify potential cardioprotective therapeutic candidates [11]. Despite this targeted approach and over 40 years of effort and investment, preclinical assessments of prospective cardioprotective agents have failed to reduce infarct size. The critically short time frame for treating ischemia to limit functional decline make it of paramount importance to develop novel therapeutics that can be delivered during the ischemic event, whether its cause is occlusive or surgical, to reduce the acute injury and prevent subsequent cardiac dysfunction. The ideal intervention should also be effective irrespective of ongoing coronary artery disease, gender, and associated comorbidities observed in these patients.

Numerous studies have shown that reducing inflammation, promoting angiogenesis and/or grafting cells during the early stage of injury can reduce adverse remodeling and cardiac dysfunction [12–15]. Recent evidence also suggests that manipulating the cell signaling that regulate apoptosis, inflammation, autophagy and bioenergetics may enhance cardiomyocyte survival and ultimately preserve function following MI [16–21]. During an ischemic event, the oxygen deficit increases the demand for anaerobic production of ATP. This is met through a combination of glycogenolysis and glycolysis. However, the capacity of cardiomyocytes to store and mobilize glycogen is finite, so most of the regulatory potential lies in modulation of glucose uptake via translocation of GLUT4 receptors to the plasma membrane [22–26]. Autophagy, a constitutive process that preserves contractile function through clearance of damaged, dysfunctional, or aged proteins, is an important pro-survival mechanism of terminally differentiated cardiomyocytes during starvation or hypoxic stress [27]. Autophagy and tolerance to oxidative stress can be modulated by sex steroids and age, and dysregulation of these processes leads to chronic inflammation and increased susceptibility to age-related diseases [28, 29]. Although considerable research has been done to find a means by which to reduce acute ischemic injury, none have yet reached the clinical realm.

The Eph receptors (Eph-R) and their ligands, the ephrins, are the largest family of membrane-bound receptor tyrosine kinases. Ephrin-Eph-R signaling regulates a variety of cell functions, ranging from cell differentiation, proliferation, and migration during development [21, 30] to tumorigenesis [31–34]. EphrinA1 is an angiogenic protein stimulated by pro-inflammatory cytokines [21, 35, 36]. Our lab and others have reported that ephrinA1-EphA-R signaling can be manipulated to salvage cardiomyocyte structure and function after MI [37, 38]. Specifically, we have previously shown that intramyocardial administration of chimeric ephrinA1-Fc limits tissue damage following a non reperfused myocardial infarction (MI) in mice and that this is partially mediated through the EphA2-R [37]. Treatment with EphrinA1-Fc also increased pAkt/Akt, decreased PARP cleavage, and increased BAG-1 expression, coordinately indicative of improved cellular survival, likely via reduced apoptosis and enhanced autophagy [37].

Based on these studies, we hypothesized that ephrinA1, normally expressed on healthy cardiomyocytes, will alter EphA-R expression in a manner conducive to improved cell survival and metabolic efficiency during ischemia, thus leading to reduced tissue damage and preservation of cardiac function. Further, we predicted that ephrinA1-Fc will reduce injury due to imbalanced substrate utilization, increased inflammation, and lipotoxicity, thus decreasing cardiomyocyte vulnerability to injury. Our results demonstrate a clear and robust cardioprotective role for exogenous EphrinA1-Fc administration during acute MI. We conclude that ephrinA1-Fc presents a novel therapeutic approach for the treatment of acute MI.

## Methods

### Animals

Experimental research protocols were fully reviewed and approved by the East Carolina University Institutional Animal Care and Use Committee (IACUC) following the guidelines of the National Institutes of Health for the Care and Use of Laboratory Animals. B6129SF2/J male mice (stock #101045) (WT) were used. Female mice were not included in this study due to confounds associated with the known cardioprotective effects of estrogen and potential interactions with EphA-Rs [39, 40]. All mice were housed in ventilated cages and animal care was maintained by the Department of Comparative Medicine at The Brody School of Medicine, East Carolina University. Mice were exposed to 12h/12h light/dark cycle conditions and received food and water ad libitum.

### Surgical procedures, cardiac function, blood and tissue collection and analyses

Male WT mice (8–12 weeks) were anesthetized with an intraperitoneal injection of 20 μl/g body weight tribromoethanol (20 mg/ml) and mechanically ventilated. Surgical procedures for coronary artery occlusion, reperfusion, blind injections of recombinant EphrinA1-Fc (E9902 Sigma) (6μg/6μl), or recombinant IgG-Fc (110 HG R&D) (6μg/6μl) as control into the border zone, the distribution of the injectate zone, closure, and recovery are described in detail elsewhere [37, 41]. Intramyocardial injection was the chosen route of administration to ensure delivery to the heart and to demonstrate the low quantity needed to achieve an effect. The injectate has not been found in lung, liver, or kidney. To minimize animal suffering and distress, two drops of 0.5% Marcaine were applied to the muscle before suturing the skin closed. Normal grooming and mobility habits of all animals were monitored every hour for the first 8 hours post-operatively and the four hours preceding echocardiography and euthanasia. The animals in the 4 day reperfusion group were also monitored daily in between surgery and at time of euthanasia at the end of the experiment by laboratory personnel specially trained in animal handling procedures by the Department of Comparative Medicine. All cages were marked with a “watchdog” card to facilitate special attention by the animal care facility staff. Of the 88 animals used in this study, all 11 animals that died during the first 24hrs post-operatively (6 in 24hr group and 5 in 4 day group) were found dead the morning after the procedure, presumably as a result of an arrhythmia as there were no other visible causes of death at the time of necropsy.

Echocardiography was performed blindly at mid-papillary level (evidenced by the presence of brush strokes at systole) on conscious, restrained mice at 24hrs or 4 days post-MI. A VisualSonics Vevo 2100 diagnostic ultrasound, using M-mode and 30MHz probe, was used to obtain LV dimensions in diastole and systole using standard procedures and calculations as previously described [42, 43].

Following echocardiography, the mice were anesthetized with a lethal intraperitoneal injection of 0.1mL pentobarbital (390mg/mL). At the time of sacrifice, a pneumothorax was performed and blood was drawn by performing a right ventricular cardiac stick and spun down to remove serum for subsequent cytokine array or ELISA analysis. Briefly, 20ul of serum from each of 10 animals per group were combined for each of the 3 groups in the 30minI/24hrR group and the proteome profiler mouse cytokine array kit, panel A (ARY006, R&D Systems, Inc) was used according to the manufacturer’s protocol. Based on the results of that exploratory experiment, ELISAs for G-CSF, KC, and BLC (Life Technologies; EMCSF3, EMCXCL1, and EMCXCL13 respectively) were run on 3 samples/group in duplicate for quantitative analysis of these cytokines accordingly to manufacturer protocol.

To assess infarct size, the heart was removed and the left ventricle was either retrogradely perfused for pthalocyanine blue/TTC staining according to routine procedures [44] and measure were made blindly using ImageJ. Other hearts were subsequently fixed in Zn-based fixative for immunohistochemical analyses as described previously [37, 43] using Ly6G (BD Pharmingen #550291) or CD45 (BD Pharmingen #550539). In each of the 4 sections from 5 hearts per group, 1 image was taken at 40x and the total number of positive cells was determined by 2 independent, blind observers and the data are represented an average of those 4 counts. For TUNEL counts, In Situ Cell Death Detection Kit (Roche, POD #11684817910) was used for staining of apoptotic cells. TUNEL+ cardiomyocyte counts are expressed as a percentage of total cardiomyocyte nuclei in an average of 3 sections in each of 3 hearts from each group. No counts were recorded in control animals since there are no positive cardiomyocyte nuclei in uninjured hearts.

Another cohort of left ventricular tissues were snap frozen for RNA for gene expression analyses or for protein extraction to conduct proteomic and western blotting analyses. Specifically, whole left ventricles of uninjured controls and I/R hearts were homogenized in Reagent 4 lysis buffer (Sigma) containing 1% protease and 1% phosphatase inhibitors. The Bradford Assay was used to quantify the amount of protein. 200ug of total protein comprised of 40ug from each of the 5 samples per group were pooled and sent to the Mass Spectrometry Laboratory at the University of Texas Health Science Center in San Antonio, Tx for proteomic analysis. Briefly, 75ug of each of the 3 pooled samples were run on a BioRad Criterion XT MOPS 12% SDS-PAGE reducing 1-D gel and stained with blue silver stain. Six slices of the bands from 10-250kDa were cut for extraction and subsequent identification using an HPLC-ESI-MS/MS on a ThermoFisher LTQ Orbitrap Velos. The data were reported using Scaffold software and following DAVID, GO, KEGG, and TRANSFAC analyses, targets were selected for validation by Western blotting. The protein concentration in each sample was determined using the Bio-Rad Bradford protein assay kit (Product #1856210). The samples were mixed with 5X loading dye, heated at 95°C for 5 mins, and then centrifuged briefly before loading. 40 μg of sample proteins were loaded in each lane and proteins were then transferred to Bio-Rad Immuno-blot polyvinylidene difluoride (PVDF) membranes (Fisher, Product #PV4HY00010). Blots were developed using the SuperSignal West Pico Chemiluminescent Substrate (Thermo Scientific, Product #34080) or SuperSignal West Femto Maximum Sensitivity Substrate (Thermo Scientific, Product #34095). Western blotting was performed using probes for Bcl-2 (3498S), Bax (2772S), mTOR (2972S), p-mTOR (2971S), LC3 (12741S), and GAPDH (2118S) from Cell Signaling. Pdk2 (PA35376), Pgam2 (PA5-24006), CD36 (MA5-14112), HSP20 (PA5-26634), and MCD (PA5-22081) from ThermoFisher and were used as per manufacturer recommendations (1:500–1000).

### Statistics

Data was stored in Microsoft Excel files and analyzed using GraphPad Prism 3 (InStat3) software. A one-way ANOVA with Student-Newman-Keuls multiple comparisons post-hoc test for the majority of analyses conducted. T-test were used when only IgG-Fc and ephrinA1-Fc groups were compared (eg. TUNEL). All data was reported as the mean ± standard deviation. These analyses were used to generate p values and Excel was used to generate the figures.

## Results

### Survival

There were no significant differences weight loss or survival in the 30minI/24hrR groups (ephrinA1-Fc: 28/32 = 88%; IgG-Fc: 29/31 = 94%). In the 30minI/4 days R groups, survival of the IgG-Fc-treated group was 71% (n = 10/14) whereas 91% of the ephrinA1-Fc-treated mice survived (n = 10/11; NS) and there were no differences in post-operative weight loss between groups at either time point.

### Echocardiography

**Fig 1:**
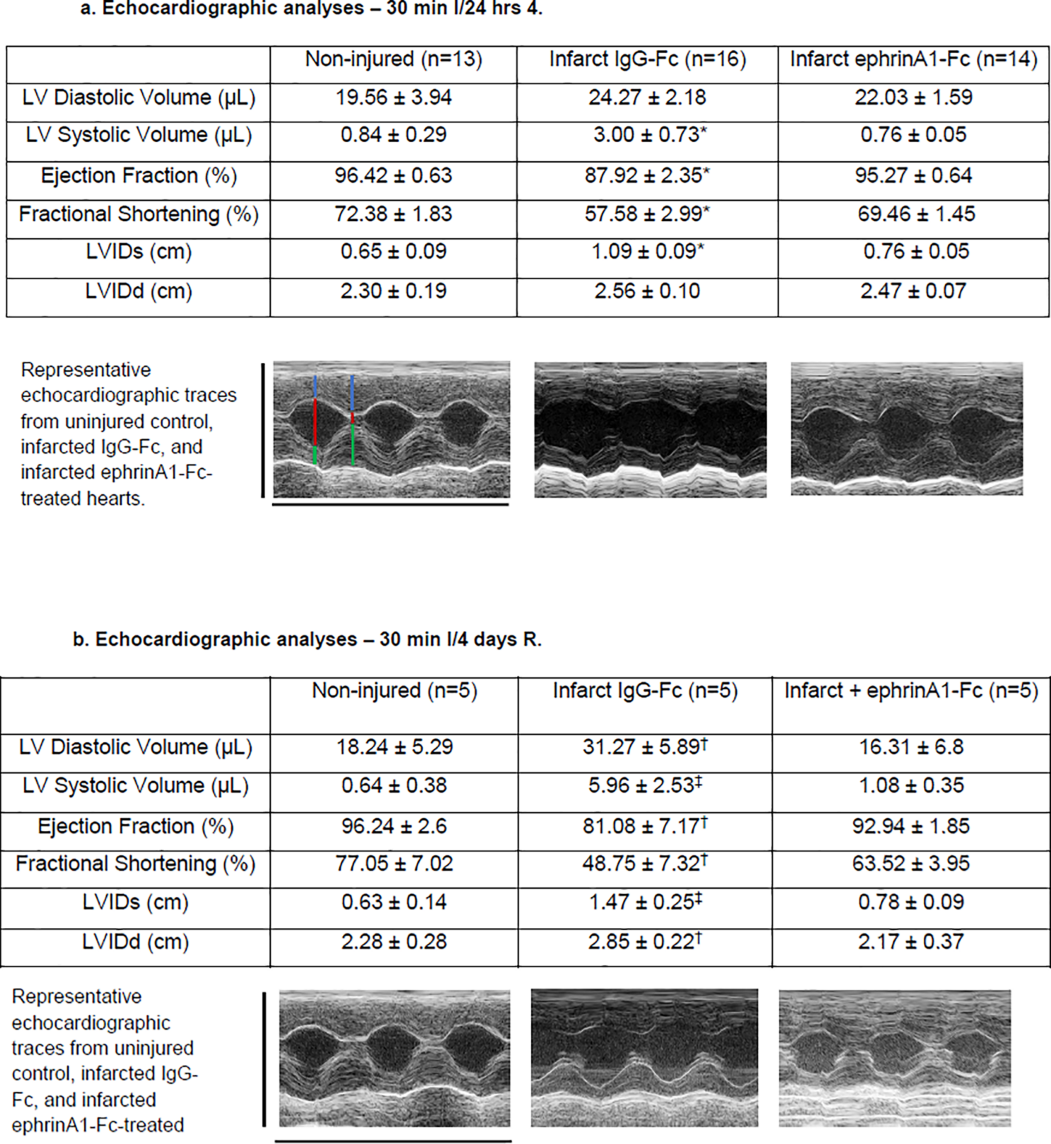
**Echocardiographic data and representative M-mode traces for uninjured controls, IgG-Fc-treated, and ephrinA1-Fc-treated mice that have undergone with 30 min of ischemia followed by 24 h reperfusion (1a) or 30 min ischemic followed by 4 d of reperfusion (1b).** Fractional shortening (FS) (57.58 ± 2.99% ^†^p < 0.01) and ejection fraction (EF) (87.92 ± 2.35%; *p < 0.05) was significantly impaired in IgG-Fc-treated mice and systolic volume and diameter were increased. In contrast, mice treated with ephrinA1-Fc showed no change in FS (69.46 ± 1.45%; p > 0.05) or EF (95.27 ± 0.64%; p > 0.05). After four days of reperfusion, infarcted IgG-Fc-treated animals display further impairment in FS (48.75 ± 7.32%; ^†^ p < 0.01) and EF (81.08 ± 7.17%; ^†^ p < 0.01) and both systolic and diastolic diameters and volumes were significantly different from uninured control and ephrinA1-Fc-treated mice whereas ephrinA1-Fc-treated animals showed no significant difference in FS (88.2 ± 5.5%; p > 0.05) or EF (96.6 ± 1.9%; p > 0.05). There were no differences in heart rate observed between the groups (control: 598 ± 64, IgG-Fc: 638 ± 69, ephrinA1-Fc: 630 ± 36) at 24hrs or 4days post-injury (control: 577 ± 53, IgG-Fc: 586 ± 57, ephrinA1-Fc: 563 ± 44). From left to right in the sham heart, blue lines denote anterior wall thickness, red lines denote chamber diameter, and green lines denote posterior wall thickness at diastole and systole, respectively.

Echocardiographic data and representative M-mode traces for uninjured controls, IgG-Fc-treated, and ephrinA1-Fc-treated mice that have undergone either 30 min of ischemia followed by 24 h reperfusion or 30 min of ischemia followed by 4 d of reperfusion are shown in Fig 1A and 1B respectively. Analysis of echocardiographic traces in IgG-Fc-treated animals 24 hours post-MI identified a significant impairment in fractional shortening (FS) (57.58 ± 2.99% *p < 0.01) and ejection fraction (EF) (87.92 ± 2.35%; *p < 0.01) compared to uninjured control mice. In contrast, mice treated with ephrinA1-Fc showed no change in FS (69.46 ± 1.45%; p > 0.05) or EF (95.27 ± 0.64%; p > 0.05). Four days post-MI, IgG-Fc-treated animals display further impairment in FS (48.75 ± 7.32%; ^†^ p < 0.001) and EF (81.08 ± 7.17%; ^†^ p < 0.001) whereas ephrinA1-Fc-treated animals showed no significant difference in FS (63.52 ± 3.95%; p > 0.05) or EF (92.94 ± 1.85%; p > 0.05). There were no differences in heart rate observed between the groups at 24hrs (control: 598 ± 64, IgG-Fc: 638 ± 69, ephrinA1-Fc: 630 ± 36) or 4 days post-injury (control: 577 ± 53, IgG-Fc: 586 ± 57, ephrinA1-Fc: 563 ± 44).

### Infarct size

**Fig 2:**
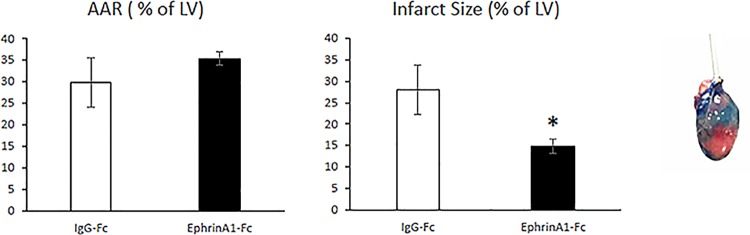
Area at risk and infarct size. Area at Risk (AAR) in IgG-Fc-treated hearts was not different from ephrinA1-Fc-treated hearts whereas infarct size was 46% smaller in the ephrinA1-Fc-treated group (*p< 0.05) compared to the IgG-Fc group.

Perfusion of excised hearts with TTC/pthalocyanine blue to differentiate viable, infarcted, and at risk regions of the tissue revealed a marked drop in necrotic tissue as a percentage of the area at risk in ephrinA1-Fc-treated mice (14.8 ± 4.6%) compared to IgG-Fc-treated mice (28.0 ± 16.3) (Fig 2). There was no difference in area at risk between ephrinA1-Fc-treated animals (35.4 ± 4.5) and IgG-Fc-treated animals (29.8 ± 15.7).

### Apoptosis and inflammatory cell infiltrate

Quantification of TUNEL staining in three tissue sections in the infarcted apical region showed a significant reduction in TUNEL+ nuclei in ephrinA1-Fc-treated animals (62.7 ± 2.4%) compared to ephrinA1-Fc-treated animals (42.3 ± 5.3%).

**Fig 3:**
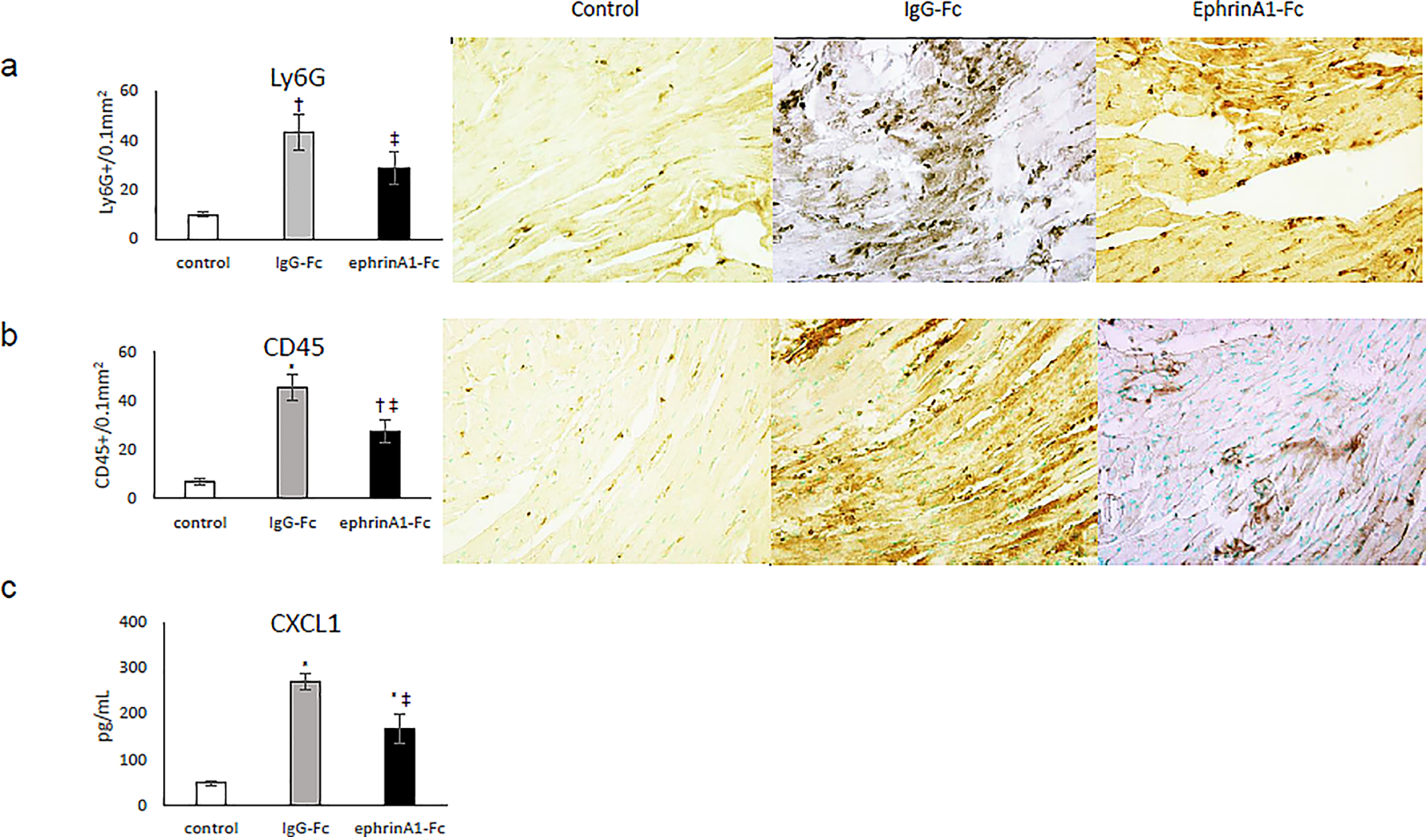
Inflammatory infiltrate in the infarct zone of IgG-Fc and ephrinA1-Fc treated mouse hearts and serum CXCL1 at 24hrs. The density of positively stained (A) Ly6G+ neutrophils and (B) CD45+ macrophages (methyl green stained nuclei completely surrounded by dark brown DAB staining) was decreased by 33% and 40% respectively in ephrinA1-Fc-treated compared to IgG-Fc-treated hearts. (C) CXCL1 in serum of ephrinA1-Fc-treated mice was significantly decreased by 39% compared to IgG-Fc-treated mice. *p<0.001 compared to control, ^†^p<0.01 compared to control, ^‡^ p<0.05 compared to IgG-Fc.

Average number of infiltrating cells/0.1mm^2^ (methyl green nuclei completely surrounded by dark brown DAB staining) and representative images for each group are shown in Fig 3. Ly6G+ cell density counts in the infarct zone of IgG-Fc-treated mice were 43 ± 7 but only 29 ± 7 in ephrinA1-Fc-treated hearts (control: 10 ± 1) and CD45+ cells were 45 ± 5 in the infarct zone of IgG-Fc-treated hearts versus only 27 ± 5 in ephrinA1-treated hearts (control: 7 ± 1).

### Cytokine array and ELISA

3 antibody arrays, one for each group, were used to obtain relative levels of serum cytokines. Of the 40 cytokines on the array, 12 cytokines were elevated in serum of both IgG-Fc and ephrinA1-Fc-treated animals (BLC, C5/C5a, G-CSF, IL-16, IL-1ra, IL-1α, MCP-1, KC, M-CSF, SDF-1, sICAM-1, and TIMP-1), but only 3 were different between the groups. Specifically, in ephrinA1-Fc-treated mice there was a 15% decrease in BLC, a 30% decrease in G-CSF, and a 35% decrease in KC compared to IgG-Fc-treated mice. ELISAs for BLC, G-CSF, and KC were used to determine the quantitative differences between groups in 3 mice/group. Results from these assays show that the cytokine with the largest differential relative expression in the cytokine array, CXCL1, was the only one with measurable difference by ELISA. Specifically, KC in IgG-Fc-treated mice was increased 5-fold but only 3-fold in ephrinA1-Fc-treated (Fig 3C). While there was an insignificant trend for G-CSF to be 30% higher in IgG-Fc-treated mice, there was no detectable difference in BLC.

### Proteomics and Western blotting

**Fig 4:**
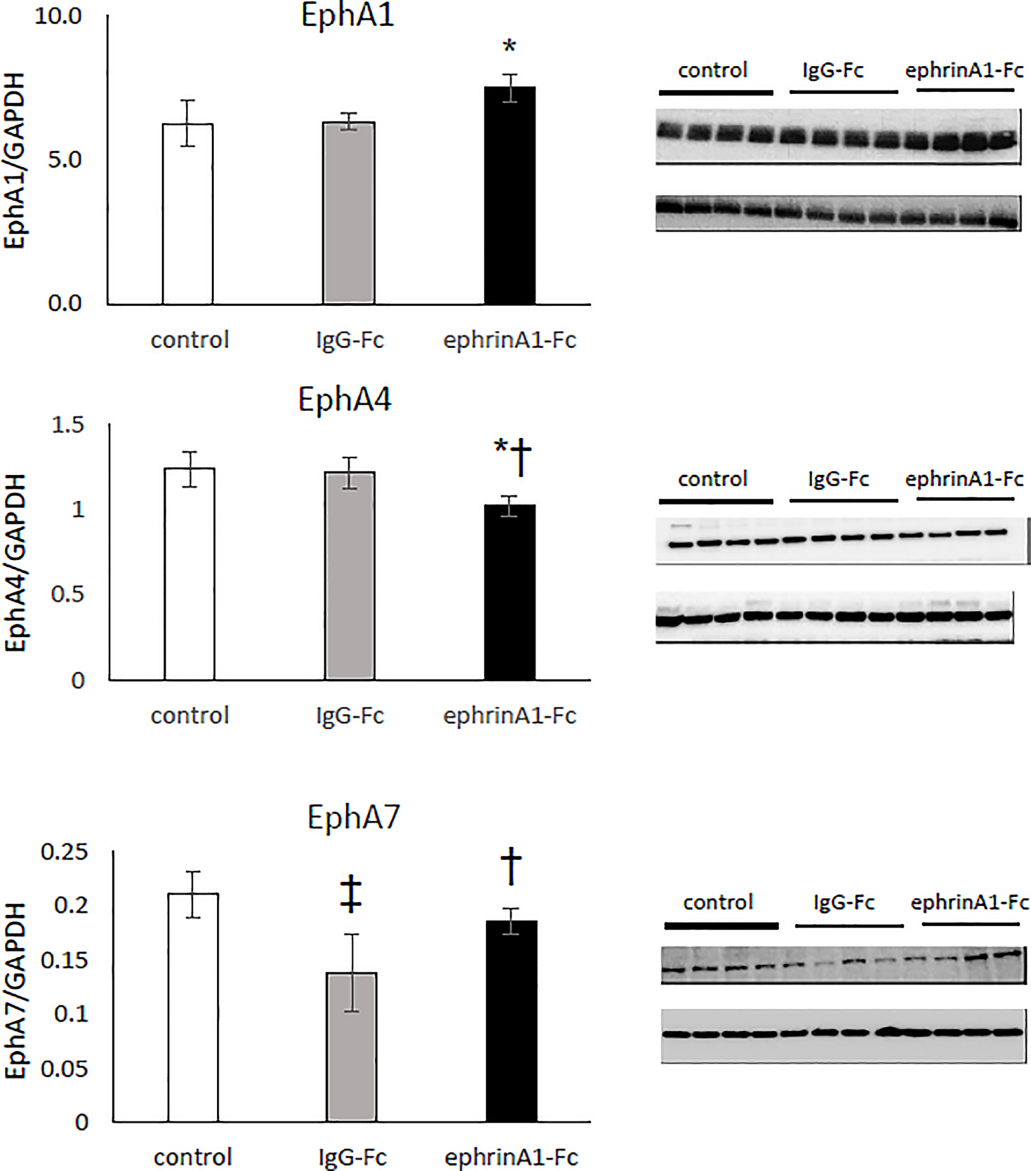
Western blots of EphA1, EphA4, and EphA7 receptor expression in left ventricular homogenates of control, IgG-Fc-treated, and ephrinA1-Fc-treated mouse hearts at 24hrs. (a) EphA1-R expression increased by 16% in ephrinA1-Fc-treated hearts compared to control hearts but were not different from IgG-Fc-treated hearts. (b) EphA4-R protein expression decreased in ephrinA1-Fc-treated hearts compared to IgG-Fc-treated hearts which were not difference from controls. (c) EphA7-R expression decreased 33% in IgG-Fc-treated hearts compared to controls and was 26% higher in ephrinA1-Fc-treatred hearts compared to IgG-Fc-treated hearts but not significantly different from control hearts. *p<0.05 compared to control, ^†^ p<0.05 compared to IgG-Fc, ‡ p<0.01 compared to control.

Fig 4 panels a-c show expression levels (normalized to GAPDH) of EphA1, EphA4, and A7 respectively in uninjured controls, IgG-Fc-treated, and ephrinA1-Fc-treated hearts after 30min/24hr (n = 4-5/group). These data indicate that there was a 16% increase in EphA1 protein expression (7.51 ± 0.49 vs 6.33 ± 0.3) and a 19% decrease in EphA4 (1.21 ± 0.09 vs 1.02 ± 0.06) in ephrinA1-Fc-treated mouse hearts compared with IgG-Fc-treated as well as uninjured control mouse hearts. In contrast, there was a 34% decrease in EphA7 (0.14 ± 0.04 vs 0.19 ± 0.01) in IgG-Fc- mouse hearts compared to ephrinA1-Fc-treated mouse hearts which remain nearly equivalent to uninjured controls.

**Fig 5:**
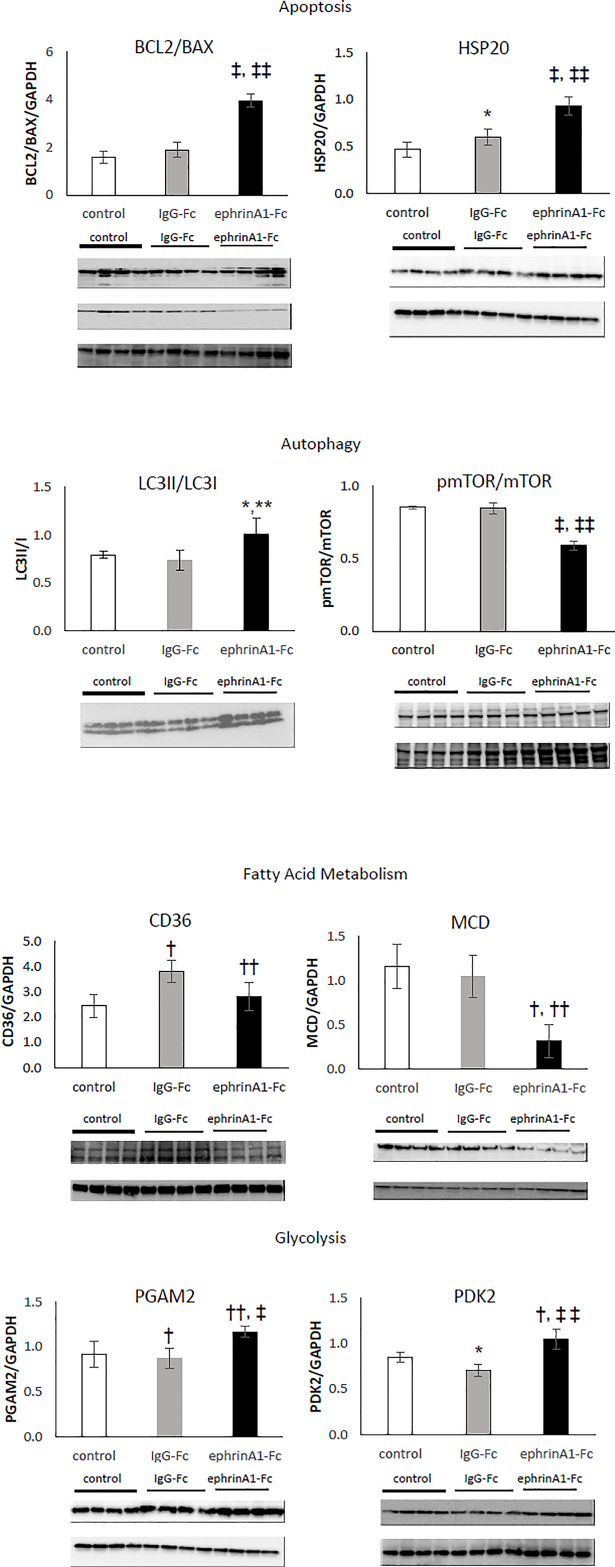
Western blots of markers for apoptosis, autophagy, fatty acid metabolism, and glycolysis in left ventricular homogenates of control, IgG-Fc-treated, and ephrinA1-Fc-treated mouse hearts at 24hrs. (a)The ratio of bcl2/bax increased 2-fold and HSP20 increased by 35%, both of which are indicative of reduced apoptosis in ephrinA1-Fc treated mouse hearts compared to IgG-Fc. (b) The ratios of LC3II/LC3I increased 28% and pmTOR/mTOR decreased by 31%, indicating increased autophagy in ephrinA1-Fc relative to IgG-Fc-treated mouse hearts. (c) Decreased CD36 and MCD in ephrinA1-Fc-treated mice by 35% and 70% respectively compared to IgG-Fc treated mice is suggestive of reduced deleterious fatty acid accumulation and increased (d) PGAM2 and PDK2 by 26% and 33% respectively indicate altered glycolytic flux. *p<0.05 compared to control, ** p<0.05 compared to IgG-Fc, ^†^ p<0.01 compared to control, †† p<0.01 compared to IgG = Fc, ‡ p<0.001 compared to control, ‡‡ p<0.001 compared to IgG-Fc.

As described above, proteomic analysis was performed on Reagent 4 extracted proteins that were run on 2-DE gels and subsequently run on an HPLC-ESI-MS/MS on a ThermoFisher LTQ Orbitrap Velos. Scaffold software was subsequently used for protein identification and changes were expressed as a ratio of ephrinA1-Fc/IgG-Fc were ranked. Antibodies against BCL2, BAX, HSP20, LC3, p-mTOR, mTOR, CD36, MCD, PDK2, and PGAM2 were selected based on the magnitude of the relative difference and pathway analysis as discussed in the methods. Graphs depicting the densitometric values of the average ± SD for n = 4-5/group and representative Western blots are shown in Fig 5A–5D.

In Fig 5A, BLC2/BAX ratio increased inephrinA1-Fc-treated hearts more than 2-fold relative the IgG-Fc-treated mouse hearts (3.96 ± 0.29 and 1.9 ± 0.31 respectively). Further, cardioprotective HSP20 (HSPB6) expression was increased by 35% in ephrinA1-Fc treated LV as compared with IgG-Fc treated hearts (0.60 ± 0.08 and 0.93 ± 0.10 respectively).

In Fig 5B, the ratio of LC3II/I was increased 27% in ephrinA1-Fc-treated mouse LV compared to IgG-Fc-treated hearts (0.74 ± 0.03 and 1.01 ± 0.17 respectively). Similarly, p-mTOR/mTOR ratio was decreased in ephrinA1-Fc-treated hearts compared to IgG-Fc-treated hearts (0.59 ± 0.03 and 0.85 ± 0.04 respectively) by 44%.

Fig 5C, CD36, an important intermediary of lipid transport across the plasma membrane into cardiomyocytes, was decreased by 35%, from 3.82 ± 0.46 in IgG-Fc-treated mice to 2.82 ± 0.57 in ephrinA1-Fc-Treated mice. Representative Western blot for MCD, a major regulator of β-oxidation, shows that there was a 233% decrease in MCD expression in ephrinA1-Fc-treated hearts compared to IgG-Fc-treated hearts from1.05 ± 0.24 to 0.31 ± 0.19.

In Fig 5D, representative Western blots for PDK2 and PGAM2, important enzymes in the glycolytic pathway that regulate ATP production, are both increased. EphrinA1-Fc treatment increased PGAM2 by 15% (1.25 ± 0.05 and 1.06 ± 0.05 respectively) and PDK2 by 33% (1.05 ± 0.11 and 0.7 ± 0.07 respectively) compared with IgG-Fc treatment.

## Discussion

Cardiomyocytes are terminally differentiated, post-mitotic cells, which lack the ability to regenerate the infarcted region following MI. Infarct size reduction and preservation of function following an ischemic insult requires cumulatively synchronized preservation of several signaling pathways. Dysregulation of metabolic function during hypoxia leads to cellular damage, resulting in release of recruitment signals into the circulatory system. Structural remodeling of the myocardial architecture in both the infarcted area and non-infarcted area ensues, leading to irreversible cardiomyocyte damage, progressive ventricular dilation, and a decline in systolic function that ultimately leads to heart failure [4–7]. Simultaneous alteration of signaling pathways that influence inflammation, apoptosis, autophagy, and cellular bioenergetics may enhance cardiomyocyte survival post-MI ischemia [16–21, 45–47]. EphrinA1, stimulated by pro-inflammatory cytokines, is an angiogenic protein that is expressed in the cardiomyocyte plasma membrane and an intramyocardial administration of chimeric ephrinA1-Fc in mice at the time of injury attenuates damage post-MI [21, 35–37, 43, 48–50]. In the present study, using a clinically relevant model of acute ischemia/reperfusion in mice, our differential staining with TTC/pthalocyanine blue data show that infarct size was reduced by 46% and there was *complete* preservation of cardiac function comparable to that of uninjured control mouse hearts at both 24hrs and 4 days post-reperfusion. Of note, in the IgG-Fc-treated infarcts, systolic function was significantly impaired at 24hr post-MI but diastolic function was not yet affected. After 4 days of reperfusion however, diastolic function was also compromised, findings which are in accordance with previous studies demonstrating time-dependent manifestation of functional changes [51, 52].

In the acute phase of ischemic myocardial injury, the release of cytokines from the infarct region signals the recruitment of immune cells to initiate the immune response to the site of injury. Quantification of Ly6G+ neutrophils and CD45+ pan-leukocytes via immunohistochemical staining of in the infarct zone of LV tissue sections indicates a robust increase in inflammatory cell recruitment in injured animals treated with IgG-Fc compared the uninjured controls. However, ephrinA1-Fc administration was associated with a significant reduction in the number of infiltrating inflammatory cells in the infarcted region at 24 hours post-MI. Attenuation of the inflammatory response to injury is further corroborated by a significant decrease in pro-inflammatory cytokines such as B lymphocyte chemoattractant (aka BLC or CXCL13), granulocyte colony-stimulating factor (G-CSF), and chemokine ligand-1 (aka KC, CXCL1, or IL-8) in the serum of ephrinA1-Fc-treated mice compared to the IgG treatment group. These chemokines are chemoattractant factors that recruit and activate neutrophils and B cells. B cells produce chemokines that elicit mobilization of monocytes to home to the site of injury, thus increasing inflammation and injury [53]. IL-8 is a potent pro-inflammatory cytokine that induce accumulation and activation of neutrophils, exacerbating tissue damage and in humans, this correlates to worse prognosis following PCI in STEMI patients due to reperfusion-related injury [54–56]. G-CSF enhances proliferation, differentiation, survival, and pro-inflammatory function of neutrophils and works synergistically with IL-8 to intensify neutrophil mobilization [57, 58]. In the present study, reduction of these cytokines in the circulation following ephrinA1-Fc treatment in I/R mice suggests that there is reduced injury and thus diminished production of homing factors. While statistically significant confirmation could only be obtained for KC by ELISA, the reduction in ischemic injury and concomitant inflammatory response point clearly to the robust cardioprotective potential of ephrinA1-Fc.

As further evidence to support of the cardioprotective properties of ephrinA1-Fc, we observed less apoptotic cardiomyocyte nuclei in ephrinA1-Fc-treated mouse hearts. Additionally, the ratio of the anti-apoptotic protein, BCL2, to the pro-apoptotic, BAX, is a key determinant in the induction of apoptosis [59]. Relative to IgG-Fc-treated infarcted hearts, BCL2/BAX is significantly elevated in response to ephrinA1-Fc treatment. We previously reported an increase in AKT activation related to delivery of ephrinA1-Fc in a model of permanent occlusion of the LAD [37]. This molecule has been established as a key intermediate involved in stimulation of the reperfusion injury survival kinases pathways that have been characterized in the context of ischemic preconditioning [60]. Additionally, we detected an increase in HSP20 (HSPB6), a highly expressed protein in the heart that is activated and phosphorylated by stress and has been shown to be cardioprotective through a variety of mechanisms, including inhibition of apoptosis and NF-κB-mediated proinflammatory cytokine production, Ca^+2^ regulation, and increased autophagy [61–65]. Taken together, less cardiac tissue damage subsequent to ephrinA1-Fc treatment could result from both direct cardioprotective signaling in cardiomyocytes and repulsion and/or decreased chemotaxis of inflammatory cells. Further investigation using isolated primary cells alone and in co-culture is in progress to identify the precise mechanism linking ephrinA1-Fc to the conservation of cardiomyocyte viability and reduced inflammation during I/R.

In conjunction with the observed cardioprotective effects, we measured increased EphA1, decreased EphA4, and preserved EphA7 protein expression in ephrinA1-Fc-treated mouse hearts as compared with IgG-Fc-treated controls (Fig 4). Decreased EphA1 expression is associated with increased leukocyte adhesion and extravasation [66], suggesting that increased EphA1 expression in response to ephrinA1-Fc may provide repulsive signals to circulating leukocytes, thereby preventing transmigration into the tissue. Similarly, Jellinghaus et al (2013) showed that ephrinA1-Fc increased endothelial expression of EphA4 in atherosclerotic plaques, rapidly increasing monocyte adhesion [67]. In our laboratory, decreased EphA4 receptor expression in ephrinA1-Fc-treated mouse hearts correlates with decrease macrophage infiltration. Lastly, although the role and distribution of EphA7 is less well-defined, Leslie-Barbick et al (2011) detected upregulation of proangiogenic EphA7 in studies of endothelial tubulogenesis in PEG hydrogels using laser scanning lithography [68]. Preservation of EphA7 expression in the ephrinA1-Fc-treated mouse hearts post-MI implies maintenance of vascular integrity. Aggregation of these changes suggest that inflammatory cell chemotaxis is inhibited by repulsive cues and vessel stabilization in myocardial tissue is maintained, both of which serve to coordinately preserve structure and function during I/R.

Additional western blotting validation of selected targets from proteomics analyses affirm that increased autophagy, decreased fatty acid oxidation and accumulation of toxic lipids in conjunction with increased ATP production via glycolysis may coordinately preserve tissue integrity and function (Fig 5). Autophagy, an essential intracellular catabolic process during which normal or dysfunctional proteins are recycled to maintain homeostasis, serves a pro-survival role during stressful conditions such as nutrient deprivation, hypoxia, and hyperglycemia by reallocating nutrients to vital processes [17, 69, 70]. During hypoxia and starvation in terminally differentiated cardiomyocytes, autophagy is an important, pro-survival mechanism. Cytoplasmic LC3 protein is processed and integrated into autophagosomal membranes and then fusion of the autophagosome with the lysosome results in the breakdown of the autophagosome vesicle and its contents. An increase in the ratio of LC3II/LC3I, is commonly used as an index of increased autophagy [71–75]. Our data show that ephrinA1-Fc treatment significantly increased this ratio, implying that autophagy is upregulated. Moreover, inhibition of mTOR by phosphorylation (a reduction in pmTOR/mTOR) suppresses protein synthesis and stimulates autophagy which reduces ischemic injury and improves cardiac function in T2DM [76–78]. We have measured a reduction in the ratio of pmTOR/mTOR in ephrinA1-Fc-treated mouse hearts compared to IgG-Fc-treated infarcted myocardium, further corroborating the increase in autophagy. While these data provide compelling evidence for the role of increased autophagic flux in myocardial protection afforded by ephrinA1-Fc, further investigation of the downstream mechanisms is required to maximize ephrinA1-Fc-mediated survival of cardiomyocytes.

In a healthy heart, a transporter of long-chain FAs, CD36, moves away from the plasma membrane and is replaced by GLUT4, representing a substantial shift from FA oxidation to glycolysis, which is associated with improved cardiac efficiency during ischemia [45–47]. However, an increase in FA uptake under these conditions in diabetic animals, can be attributed to increased expression of CD36, and is associated with impaired cardiac function [79]. Inhibition of CD36 has been shown to prevent lipid accumulation and detrimental effects on contractile function while preserving insulin signaling [80, 81]. Quantification of CD36 protein abundance by western blot has shown a significant drop in CD36 expression in ephrinA1-Fc-treated hearts compared to vehicle controls, suggesting reduced uptake of FAs into the cardiomyocytes after ephrinA1-Fc treatment. This observation is made in conjunction with a dramatic reduction in malonyl-CoA decarboxylase (MCD), the enzyme responsible for the conversion of malonyl-coA to acetyl-coA, which decreases cardiac FA oxidation through allosteric regulation of the mitochondrial FA transporter, carnitine palmitoyltransferase 1 (CPT1) [82, 83]. Inhibition of MCD resulting in increased malonyl-coA accumulation is associated with increased glucose oxidation and cardioprotection from ischemia [82, 84–90]. Studies are underway to evaluate ephrinA1-Fc-mediated substrate preference and energy production.

Changes in glucose oxidation correlate to both the duration of the ischemic event and the substrates provided during reperfusion. Inclusion of fatty acids along with glucose has been shown to decrease responsiveness compared to use of glucose as the sole substrate. Increasing pyruvate has also been shown to improve the mechanical performance of the heart. Pyruvate dehydrogenase kinase isozyme 2 (PDK2), a major regulator of the pyruvate dehydrogenase complex (PDC), is highly expressed in the heart and play a pivotal role in coordinating the balance between glucose and FA metabolism [91]. Western blot analysis of ephrinA1-Fc-treated hearts shows that PDK2 protein increased, likely resulting in increased negative regulation of PDC which subsequently attenuated aerobic catabolism by shunting glucose-derived pyruvate towards the formation of lactate. This is important in tissues with high ATP requirement.

Phosphoglycerate mutase 2 (PGAM2) is a glycolytic enzyme known to be expressed in anaerobic tissues including skeletal muscle and cancers cells. Increased oxidative stress in tumor cells stimulates PGAM2 activity which enhances glycolytic flux (the Warburg effect), enabling cells to adapt to hypoxic conditions [92]. Deficiency of PGAM2 in muscle is associated with intolerance for strenuous exercise [93]. Although persistent overexpression in mouse heart altered mitochondrial respiratory capacity and ROS generation resulting in less tolerance to stress [94], ephrinA1-Fc treatment resulted in an increase in PGAM2, suggesting that acute and/or transient upregulation is metabolically beneficial. Although we have not yet verified differences in expression levels or performed substrate utilization assays, the mass spectrometry results from pooled samples indicate that lactate dehydrogenase, enolase 1,pyruvate dehydrogenase, and mitochondrial pyruvate carrier are all increased, whereas CPT1 is decreased and CPT 2 and carnitine acetyltransferase (CrAT) are increased, providing further evidence that ephrinA1-Fc causes shunting of substrates away from oxidation of fatty acids to oxidation of glucose and lactate, leading to improved ATP production and reduced tissue damage [95–102].

Collectively, these results defy conventional beliefs regarding the fragility of highly oxygen-sensitive, terminally-differentiated cardiomyocytes and the mechanisms by which ischemia can be acutely managed.

### Conclusions and future studies

The current investigation demonstrates the cardioprotective efficacy of ephrinA1-Fc following I/R in the adult male mouse myocardium. Real-time visualization and measurement of LV chamber dimensions in conscious mice showed significantly impaired contractile function in infarcted mice that received IgG-Fc, an effect that was rescued by the provision of ephrinA1-Fc at the time of coronary artery ligation. Specifically, our data show that changes in ephrinA1/EphA-R gene expression, serum cytokine levels and inflammatory cell infiltration, decreased apoptosis, reduced fatty acid oxidation and associated lipotoxicity, and increased glycolytic flux culminate in improved energy production and utilization efficiency, decreased myocardial infarct injury and complete preservation of cardiac function. These data clearly demonstrate a cardioprotective role for EphrinA1-Fc and may present a novel therapeutic approach in the treatment of MI.

Future studies of the effects of ephrinA1/EphA-R signaling pathways involved in apoptosis, autophagy, and metabolism in isolated in adult primary cardiomyocytes as well as intact animals of both sexes, aged mice, and administration of ephrinA1 at the time of reperfusion are in progress and will advance our understanding of the role of ephrinA1/EphA-R in attenuating ischemic injury. Results from these studies will provide a comprehensive analysis of the pathways coordinately regulated by ephrinA1/EphA-R and how they are altered by ephrinA1-Fc to effect protection of the ischemic heart. This will underscore its potential translational impact in designing customized treatment paradigms to reduce ischemic heart disease and improve myocardial performance in both genders at any age irrespective of associated co-morbidities.
